# A pilot study comparing the pharmacokinetics of injectable cyanocobalamin and hydroxocobalamin associated with a trace mineral injection in cattle

**DOI:** 10.1111/jvp.12967

**Published:** 2021-03-08

**Authors:** Paula A. Gonzalez‐Rivas, Michael Chambers, Jerry Liu

**Affiliations:** ^1^ Virbac Australia Pty Ltd Milperra NSW Australia; ^2^ School of Agriculture and Food, Faculty of Veterinary and Agricultural Sciences The University of Melbourne Melbourne VIC Australia; ^3^ Invetus Pty Ltd Armidale NSW Australia

**Keywords:** cattle, cobalamin, cyanocobalamin, hydroxocobalamin, vitamin B_12_

## Abstract

Injectable vitamin B_12_ (cobalamin) is traditionally used to prevent or treat vitamin B_12_ deficiencies in ruminants. Sheep and human studies have demonstrated the superiority of a single dose of hydroxocobalamin (OHB12) over cyanocobalamin (CNB12) in maintaining high levels of cobalamin in plasma and liver. However, limited data are available for cattle. The purpose of this study was to compare the pharmacokinetics of two forms of cobalamin—OHB12 and CNB12—as a single subcutaneous injection of 28 µg/kg BW at the same time of a trace mineral injection in six non‐cobalt/B_12_‐deficient Holstein‐Friesian steers. Plasma and liver samples were obtained to determine cobalamin concentration after treatment. Cyanocobalamin had lower retention in plasma and liver than OHB12 (p < .05). Cobalamin levels peaked in plasma by 8 h after treatment in both groups. However, OHB12 reached a higher peak compared to CNB12. Levels of cobalamin in plasma dropped closer to baseline levels 24 h after CNB12 treatment while OHB12 maintained higher concentrations. Hydroxocobalamin increased significantly hepatic concentration of cobalamin 28 days after treatment, while CNB12 did not increase liver levels relative to pre‐treatment (p < .05). These results confirm that a single subcutaneous OHB12 injection increases the level of cobalamin in the blood in the first 24 hours, and this increase is maintained in the liver for at least 28 days.

Sheep and cattle require cobalt (Co) for the synthesis of vitamin B_12_ (cobalamin) by reticulo‐rumen microorganisms (Grace and Knowles, 2012; Suttle, 2010). Under grazing conditions, sheep are more susceptible to Co/B_12_ deficiencies and have been the focus of more research on this topic compared to cattle (Grace and Knowles, 2012; Suttle, 2010). In Co‐deficient areas of Australia, such as coastal areas, calcium‐rich or sandy soils, dietary Co or injectable cobalamin supplementation are recommended to prevent or treat deficiencies (Freer et al., 2007; Suttle, 2010). In addition, there has been recent interest in developing injectable pharmaceutical products to supplement trace minerals and vitamin B_12_ for cattle. Currently, two commercially available analogue forms of injectable cobalamin, hydroxocobalamin (OHB12) and cyanocobalamin (CNB12), are registered for use in livestock. Cyanocobalamin is not the naturally occurring form of cobalamin in the animal's body; a reductive decyanation reaction is an enzymatically controlled process required to remove the cyanide group from CNB12 (Kim et al., 2008; Jeong and Kim, 2011). Only when the cyanide group is removed, CNB12 is converted to OHB12, a common intermediate, which is then transformed in the body into methylcobalamin or deoxyadenosylcobalamin (5‐deoxyadenosylcobalamin), the physiologically active forms or coenzymes of vitamin B_12_ (McDowell, 2000; Kim et al., 2008; Paul and Brady, 2017). Although CNB12 is the most widely used form of cobalamin in pharmaceutical preparations because of its chemical stability, OHB12 is better retained after parenteral administration than CNB12 (McDowell, 2000). Sheep studies have demonstrated the superiority of a single dose of OHB12 over CNB12 in maintaining high levels of cobalamin in plasma and liver (Judson et al., 2002). However, data comparing the different forms of cobalamin in cattle are lacking, and a clear distinction of which form of cobalamin was used is not always reported in the literature. The purpose of this study was to compare the pharmacokinetics of two analogous forms of cobalamin (OHB12 and CNB12) in cattle and determine which cobalamin form is more effective in raising plasma and liver cobalamin levels.

This study was conducted with the approval of the Virbac Animal Ethics Committee (AEC 613/20) following the Australian Code for the Care and Use of Animals for Scientific Purposes National Health and Medical Research Council (2013). The study was conducted on a commercial farm in Gippsland, Victoria, Australia, between June and September 2020.

A total of 6 Holstein‐Friesian steers (12–18‐month‐old, 385 ± 16.1 kg) were included in this pilot study. On day ‐5, animals were weighed, and liver biopsies and blood samples were obtained to determine basal levels of cobalamin in plasma and liver. Blood samples (10 ml) were obtained from the caudal vein in lithium heparin tubes (BD Vacutainer®, Becton Dickenson). On collection, the red cells were immediately separated from plasma by centrifugation to prevent any cobalamin exchanges. Liver biopsies were obtained following standard protocol under xylazine sedation (Parkinson et al., 2019).

On day 0, animals were allocated to one out of two treatment groups based on bodyweight and basal cobalamin levels. The first group (CNB12) received one subcutaneous injection of a product containing 26.7 mg Zn/ml as disodium zinc EDTA, 6.7 mg Mn/ml as disodium manganese EDTA, 3.3mg Se/ml as sodium selenite, 10 mg Cu/ml as disodium copper EDTA and 1.4 mg cyanocobalamin/ml (1.5 ml/75 kg, Marks‐Min, Boehringer Ingelheim Animal Health Australia Pty Ltd). The second group (OHB12) received one subcutaneous injection of a product containing 40 mg Zn/ml as disodium zinc EDTA, 10 mg Mn/ml as disodium manganese EDTA, 5 mg Se/ml as sodium selenite, 15 mg Cu/ml as disodium copper EDTA (1 ml/75 kg, Multimin, Virbac Australia Pty Ltd) and one subcutaneous injection of a product containing 2 mg hydroxocobalamin acetate/ml (Cobalife, Elanco Australasia Pty Ltd). All pharmaceutical products were injected as per label recommendation, and both treatment groups received the same amount of trace minerals and equivalent doses of cobalamin on a bodyweight basis (Table 1).

**TABLE 1:** Amounts of trace minerals and cobalamin and volume of commercial products injected on a bodyweight basis

Item/kg BW	CNB12		OHB12	
	Mean	SD	Mean	SD
Cu, mg	0.20	0.001	0.20	0.002
Se, mg	0.07	0.000	0.07	0.000
Zn, mg	0.54	0.002	0.54	0.004
Mn, mg	0.13	0.000	0.13	0.001
Vitamin B_12_, µg	28.0	0.08	28.0	0.05
Total volume, ml^a^	0.020	0.000	0.027	0.000

CNB12: animals (*n* = 3) received one subcutaneous injection of a product containing 26.7 mg Zn/ml as disodium zinc EDTA, 6.7 mg Mn/ml as disodium manganese EDTA, 3.3mg Se/ml as sodium selenite, 10 mg Cu/ml as disodium copper EDTA and 1.4 mg cyanocobalamin/ml (1.5 ml/75 kg BW, Marks‐Min, Boehringer Ingelheim Animal Health Australia Pty Ltd).

*OHB12:* animals (*n* = 3) received one subcutaneous injection of a product containing 40 mg Zn/ml as disodium zinc EDTA, 10 mg Mn/ml as disodium manganese EDTA, 5 mg Se/ml as sodium selenite, 15 mg Cu/ml as disodium copper EDTA (1 ml/75 kg BW, Multimin, Virbac Australia Pty Ltd) and one subcutaneous injection of a product containing 2 mg hydroxocobalamin acetate/ml (Cobalife, Elanco Australasia Pty Ltd).

^a^ Total volume injected: CNB12 = 0.020 ml/kg BW Marks‐Min and OHB12 = 0.027 ml/kg BW (0.013 ml Multimin + 0.014 ml Cobalife).

Blood samples were obtained 8 h, 24 h, 7 days, 28 days and 56 days after treatment and liver biopsies were collected 7, 28 and 56 days after treatment. Plasma and liver samples were analysed for cobalamin levels by a commercial laboratory using the modified CEDIA GenII electrochemiluminescence immunoassay (ECLIA—Roche Cobas e411; Regional Laboratory Services [RLS], Benalla, Vic, Australia). Briefly, vitamin B_12_ was measured based on intrinsic factor with streptavidin and biotin (Roche cat#07212771); modifications to the assay are as per RLS 085 Issue B, Rev 4, and include a pre‐boiling step prior to the e411 assay, to eliminate assay interferences observed in non‐boiled assays. Full details of these modifications are commercial‐in‐confidence to RLS.

Animal health status was monitored daily, and initial and final bodyweights were obtained using a walk‐over scale. The six animals were kept in a group paddock of approximately 4 hectares of perennial ryegrass with an average yield of 2250 kg DM/ha and a target intake of 10 kg DM/day per animal, with ad libitum access to water, and without feed supplements or concentrate.

Pasture samples were analysed for nutrients and Co content before the experiment (day ‐45) and once a month until the end of the experiment. Samples were sent to a commercial laboratory for analysis (Livestock Logic, Hamilton, Vic, Australia; Table 2).

**TABLE 2:** Pasture nutritional analysis

Parameter	Experimental day			
	Day −45	Day 0	Day 28	Day 56
Total dry matter (DM), %	15	14	15	16
Moisture, %	85	86	85	84
Digestible dry matter (DDM), %DM	83	80	81	79
Digestibility of organic dry matter (DODM), %DM	77	75	74	74
Metabolizable energy (ME), MJ/kg DM	12.6	12.2	12.1	12.0
Crude protein (N × 6.25) (CP), % DM	25.9	24.7	25	26.2
Neutral detergent fibre (NDF), % DM	49	59	54	55
Acid detergent fibre (ADF), % DM	23	29	27	28
Water soluble carbohydrates (WSC), % DM	7	5	5	<4
Ash, % DM	11	11	12	23
Cobalt, mg/kg DM	0.28	0.61	0.54	0.78
Yield, kg DM/ha	2000	2000	2500	2500

Data were collated by treatment group and time point. Treatment mean concentrations by time point and associated standard deviations and standard errors were calculated. Blood plasma and liver vitamin B_12_ concentrations were compared between treatments using repeated measures analysis of variance and Statistix 10.0 (Analytical Software 2013). Means were compared using Tukey’s all‐pairwise comparison test, for *Treatment*, *Time* and *Treatment* × *Time*. Model assumptions were checked via residual plots and were considered acceptable given the size of the data set (*n* = 3/group). Homogeneity of variance assumptions were checked and confirmed via Levene's test. Means and associated standard errors are presented in Table 3.

**TABLE 3:** Treatment group mean and SE of plasma and liver vitamin B_12_ concentrations by time point

Time point (Study day)	CNB12		OHB12	
	Mean	SE	Mean	SE
Plasma vitamin B_12_ (pmol/L)				
−5	255^c^	9	279^c^	17
0 (Tx + 8 hours)	3260^b^	259	4300^a^	0
1	801^c^	80	2803^b^	39
7	332^c^	10	446^c^	16
28	301^c^	20	324^c^	11
56	256^c^	13	283^c^	23
Liver vitamin B_12_ (nmol/kg)				
−5	529^b^	7	596^b^	34
7	553^b^	31	696^ab^	101
28	685^ab^	46	928^a^	83
56	601^b^	43	678^ab^	15

CNB12: animals (*n* = 3) received one subcutaneous injection of a product containing 26.7 mg Zn/ml as disodium zinc EDTA, 6.7 mg Mn/ml as disodium manganese EDTA, 3.3mg Se/ml as sodium selenite, 10 mg Cu/ml as disodium copper EDTA and 1.4 mg cyanocobalamin/ml (1.5 ml/75 kg BW, Marks‐Min, Boehringer Ingelheim Animal Health Australia Pty Ltd).

OHB12: animals (*n* = 3) received one subcutaneous injection of a product containing 40 mg Zn/ml as disodium zinc EDTA, 10 mg Mn/ml as disodium manganese EDTA, 5 mg Se/ml as sodium selenite, 15 mg Cu/ml as disodium copper EDTA (1 ml/75 kg BW, Multimin, Virbac Australia Pty Ltd) and one subcutaneous injection of a product containing 2 mg hydroxocobalamin acetate/ml (Cobalife, Elanco Australasia Pty Ltd).

^a,b,c^Means sharing the same superscript are not significantly different from each other (Tukey's HSD, p < .05).

Cobalt levels in the pasture before and during the trial were within the recommended range to prevent cobalamin deficiency in cattle (>0.10 mg Co/kg DM; Freer et al., 2007), and cobalamin levels in liver and plasma were within the reference range for cattle at day ‐5 (>200 nmol/kg wet weight and > 220 pmol/L for liver and plasma, respectively; Stangl et al., 2000; Grace et al., 2010). This confirms that cattle were not deficient in cobalamin at the start of this experiment.

Analysis of plasma and liver vitamin B_12_ concentrations demonstrated that groups were not significantly different prior to treatment and that plasma cobalamin peaked at 8 h for both groups. However, a higher peak of cobalamin in plasma was observed in animals treated with OHB12 compared to CNB12, and this difference was maintained at 24 h (p = .002). Twenty‐four hours post‐injection, the cobalamin levels of OHB12 treated animals declined, while cobalamin levels in CNB12‐treated animals were not different from basal levels. By day 7, plasma cobalamin concentrations for both groups approximated the original baseline, and no further changes were observed at days 28 and 56 post‐treatment (Table 3).

Steers receiving OHB12 had higher but not significantly different liver levels of cobalamin than baseline at day 7 (596 nmol/kg vs 696 nmol/kg). By contrast, animals treated with CNB12 showed no difference from the baseline. At day 28, steers treated with OHB12 had peak levels of cobalamin in the liver that were higher than those treated with CNB12 (928 nmol/kg vs 685 nmol/kg). Although this difference was not statistically significant in this study, it may be of clinical and productive importance. A study with a larger number of cattle would be required to confirm those differences.

Nevertheless, at day 28, steers treated with CNB12 showed no difference from baseline cobalamin liver levels. In contrast, those treated with OHB12 had significantly higher liver cobalamin relative to baseline (928 nmol/kg vs 596 nmol/kg, p < .05). By day 56, both groups had liver cobalamin levels not different from baseline (Table 3).

The average daily gain (ADG) of steers receiving OHB12 was approximately 11 % higher than that observed in those treated with CNB12 (1.17 kg /day vs 1.06 kg/day; p = .572). The ADG of > 1 kg/day demonstrates adequate nutrition for Holstein‐Friesian steers grazing improved pasture during winter in South Gippsland, Victoria. The numerically higher ADG for steers in OHB12 was not statistically significant than in those treated with CNB12. A study with a larger number of cattle would be required to demonstrate differences in ADG in pasture‐fed cattle supplemented with different forms of vitamin B_12_.

The results of this pilot study demonstrate that a single injection of OHB12 can be effective in increasing the level of cobalamin in the blood in the first 24 hours and this increase is maintained in the liver for at least 28 days in non‐Co/cobalamin‐deficient cattle, while CNB12 was unable to raise liver cobalamin reserves relative to basal levels at any time point. These results agreed with previous studies conducted in sheep and humans. The reduced response to CNB12 is based on its lower bioavailability, rapid mobilization from the injection site, and fast renal clearance resulting in a shorter duration of activity compared with OHB12 (Hall et al., 1984; Judson, 1996; McDowell, 2000; Judson et al., 2002; Paul and Brady, 2017).

Judson (1996) described that lambs given either 2 mg OHB12 or 2 mg CNB12 excreted about 45 % and 70 % of the respective doses within 6 hours of treatment and OHB12 was more effective in raising liver cobalamin reserves than CNB12. They concluded that OHB12 is the preferred cobalamin analogue to be used in sheep. In another experiment, a single injection of a solution containing 90 % OHB12 and 10 % CNB12 increased cobalamin concentration in blood above control values between 28 and 51 days in Co‐deficient animals (Judson et al., 2002). It is accepted that the protective period of an OHB12 injection is about 1–3 months in lambs depending on deficiency status (Grace et al., 1998; Judson, 1996; Judson et al., 2002). Previous studies have highlighted the differences in cobalamin metabolism between sheep and cattle, for a similar dose rate; the length of efficacy in cattle can be half that of lambs (Grace et al., 2010). A similar situation was observed in this trial where regardless of deficiency status, high levels of cobalamin in the liver were maintained for at least 28 days after OHB12 injection.

Human studies demonstrated that the rise in plasma cobalamin levels was more sustained and that urinary excretion of cobalamin was considerably lower after the intramuscular injection of OHB12 than after the injection of CNB12 (Herbert et al., 1963). Human cell studies conducted by Hall et al. (1984) demonstrated that OHB12 is an efficient form of treatment of cobalamin deficiencies because of its greater availability to cells and better retention, thereby requiring less frequent injections than CNB12. A recent review (Paul and Brady, 2017) concluded that supplementing with the natural cobalamin analogue OHB12 is preferred instead of CNB12 due to its superior bioavailability and safety in humans.

Weekly injections of CNB12 (from ~2.5 to 20 µg/kg BW) alone or in combination with butaphosphan, folic acid or other additives from 8 to 3 weeks before calving until 9 to 18 weeks of lactation were used to evaluate their effect on improving energy status, gluconeogenesis, lactation and/or preventing ketosis in different studies in dairy cows (Akins et al., 2013; Duplessis et al., 2017; Girard and Matte, 2005; Preynat et al., 2009; Weerathilake et al., 2019). In those studies, plasma and/or liver concentration of cobalamin was higher for cows receiving the CNB12 supplement than those that did not. However, no kinetic information was presented in these studies and blood and liver samples were obtained only a couple of days after the CNB12 administration. Furthermore, none of those studies reported the duration of the activity of CNB12 after a single injection of cobalamin.

The results of the current pilot study agreed with Clark et al. (1986). They demonstrated that monthly subcutaneous injections of OHB12 to non‐cobalt‐deficient heifers elevated cobalamin levels in liver above those of untreated animals. However, the responses observed in the current trial are lower than those previously observed by Judson et al. (1981) where a single injection of 2 mg of OHB12 raised blood cobalamin levels for 12 weeks in 3‐month‐old calves in a Co‐deficient area. Differences in the cobalamin metabolism after OHB12 and CNB12 supplementation in Co/cobalamin‐deficient and non‐deficient cattle will require further investigation, and more frequent sampling after treatment to determine early pharmacokinetics are guaranteed.

The current pilot study confirmed the different pharmacokinetics of two analogous forms of cobalamin (OHB12 and CNB12) in cattle. This study demonstrated that a single subcutaneous OHB12 injection increases the level of cobalamin in the blood in the first 24 hours and this increase is maintained in the liver for at least 28 days in non‐Co/cobalamin‐deficient cattle when used in combination with trace minerals and demonstrated that a single dose of CNB12 is not an effective therapeutic method to raise cobalamin levels in cattle as increased cobalamin levels in the blood were not sustained for more than 24 hours, and liver cobalamin storage levels were not elevated from baseline at any time point. The authors acknowledge that injectable cobalamin was not administered as a pure solution but associated with trace minerals which could affect animal metabolism. The experimental design did not allow the authors to infer the presence or absence of such effect, and therefore, further studies to clarify this are guaranteed. Furthermore, additional studies, including a larger number of animals and more frequent sampling after treatment, are recommended to evaluate the effect of CNB12 and OHB12 in Co/cobalamin‐deficient cattle on blood and liver pharmacokinetics and bodyweight change.

## 1 ANIMAL WELFARE AND ETHICS STATEMENT

This study was conducted with the approval of the Virbac Animal Ethics Committee (AEC 613/20) following the Australian Code for the Care and Use of Animals for Scientific Purposes, National Health and Medical Research Council (2013).

## CONFLICT OF INTEREST

Dr Paula A Gonzalez‐Rivas and Dr Jerry Liu are employees of Virbac Australia Pty Ltd. Multimin ^®^ is a registered trademark of Virbac Australia Pty Ltd.

## Author Contribution

PAGR and JL designed the experiment; PAGR supervised the execution of the experiment; MC analysed the data; and PAGR wrote the manuscript in consultation with JL and MC.

## Data Availability

The data that support the findings of this study are available from the corresponding author (PAGR) upon reasonable request.
